# A case of surgically treated non-metastatic SMARCA4-deficient undifferentiated thoracic tumor: a case report and literature review

**DOI:** 10.3389/fonc.2024.1399868

**Published:** 2024-06-06

**Authors:** Cong Yin, Zheng-jia Liu, Chao He, Hai-xiang Yu

**Affiliations:** Department of Thoracic Surgery, China-Japan Union Hospital of Jilin University, Changchun, China

**Keywords:** SMARCA4-UT, treatment of SMARCA4-UT, non-metastatic SMARCA4-UT, PET/CT, lung cancer

## Abstract

SMARCA4-deficient undifferentiated thoracic tumor (SMARCA4-UT) is a rare malignant tumor characterized by inactivation of the *SMARCA4* gene and the presence of undifferentiated or rhabdoid morphology in the tissue. This tumor is highly invasive, typically diagnosed at advanced stages III or IV, and commonly involves thoracic structures, such as the mediastinum and chest wall. Reported cases are limited and treatment guidelines have not yet been established. Here, we present a rare case of surgically treated non-metastatic SMARCA4-UT. The patient presented with blood-tinged sputum, dyspnea, and a history of heavy smoking, and underwent surgery after preoperative evaluation ruled out contraindications. The tumor was successfully removed along with the relevant lymph nodes; analysis determined it to be stage IIB T3N0M0. No recurrence was detected at two months post-surgery. However, four months after surgery, the tumor recurred and invaded the adjacent ribs. The diagnosis, differential diagnosis, and treatment of SMARCA4-deficient undifferentiated lung tumors is considered. The combination of chemotherapy and immunotherapy has shown efficacy, and other treatments such as anti-angiogenic drugs, histone deacetylase inhibitors (HDACi), enhancer of zeste 2 polycomb repressive complex 2 subunit (EZH2) inhibitors, and oxidative phosphorylation (OXPHOS) inhibitors may also be beneficial in treating SMARCA4-UT.

## Introduction

SMARCA4-deficient undifferentiated thoracic tumor (SMARCA4-UT) is a rare malignant tumor characterized by inactivation of the *SMARCA4* gene and the presence of undifferentiated or rhabdoid morphology in the tissue. Previously known as SMARCA4-deficient thoracic sarcoma (1), SMARCA4-UT is associated with smoking and displays unique clinical and pathological features (2). The World Health Organization categorizes SMARCA4-UT in the other lung epithelial tumors’ group (3). Typically diagnosed at advanced stages, SMARCA4-UT often extensively involves thoracic structures and metastasizes to the lymph nodes, bones, and adrenal glands (2, 4). Although radiotherapy and chemotherapy have limited efficacy, immunotherapy is effective in these patients (5–8). However, the lack of definitive treatment guidelines and scarcity of reported cases, especially those suitable for surgery, pose challenges in the diagnosis and management of SMARCA4-UT. Here, we present the case of a patient with a stage II tumor who underwent surgical intervention, and we discuss the diagnosis and treatment of SMARCA4-deficient undifferentiated lung tumors.

## Case presentation

**Figure 1:**
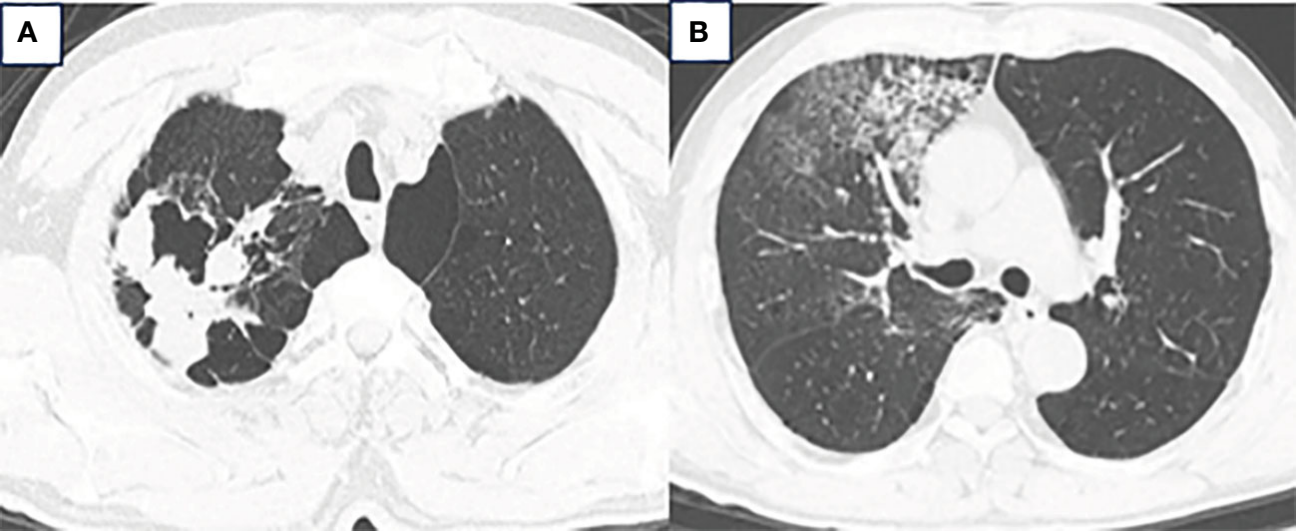
**(A)** Chest CT showed a mass in the right upper lobe measuring approximately 6.3 × 5.7 × 4.3 cm with a cavity. **(B)** Inflammation in the right upper and middle lobes.

**Figure 2:**
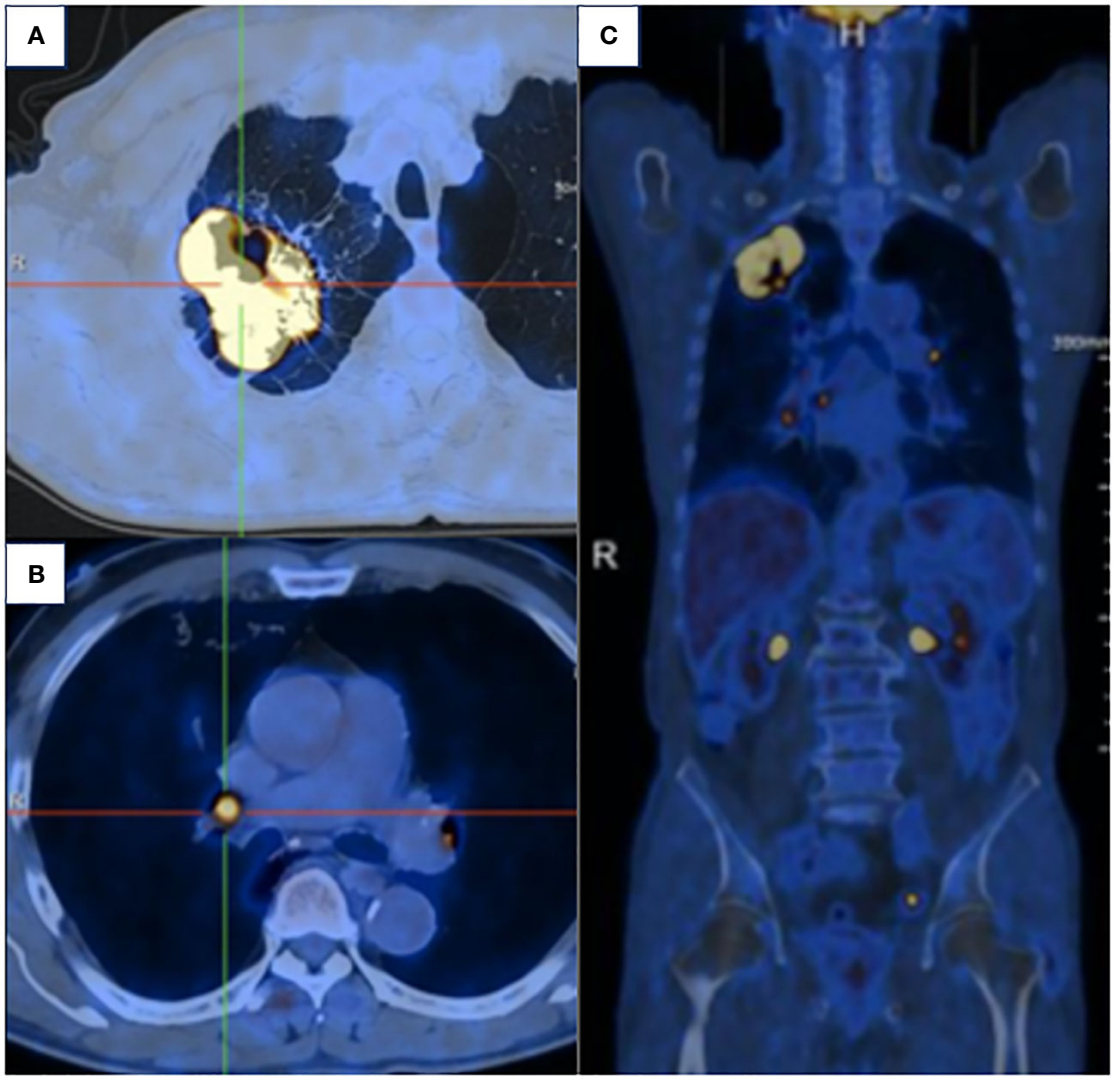
**(A)** PET/CT demonstrated the mass in the right upper lobe with a maximum SUV of 44.67. **(B)** Right lung hilar lymph node metastasis could not be excluded. **(C)** No distant organ metastasis was observed.

**Figure 3:**
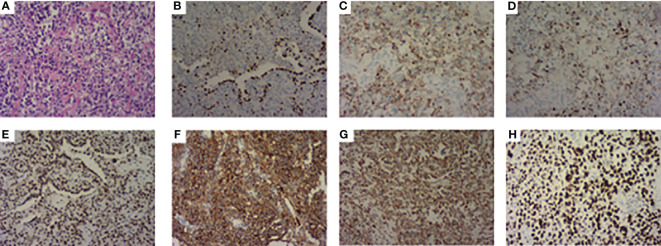
Pathological features. **(A)** Stained with hematoxylin and eosin. **(B)** Immunohistochemistry showed BRG1 is negative. **(C)** CK 19 and **(D)** CK (pan) were focally positive. **(E)** INI-1, **(F)** EMA, and **(G)** Vimentin were positive. **(H)** The proliferation index Ki-67 was 90%.

**Figure 4:**
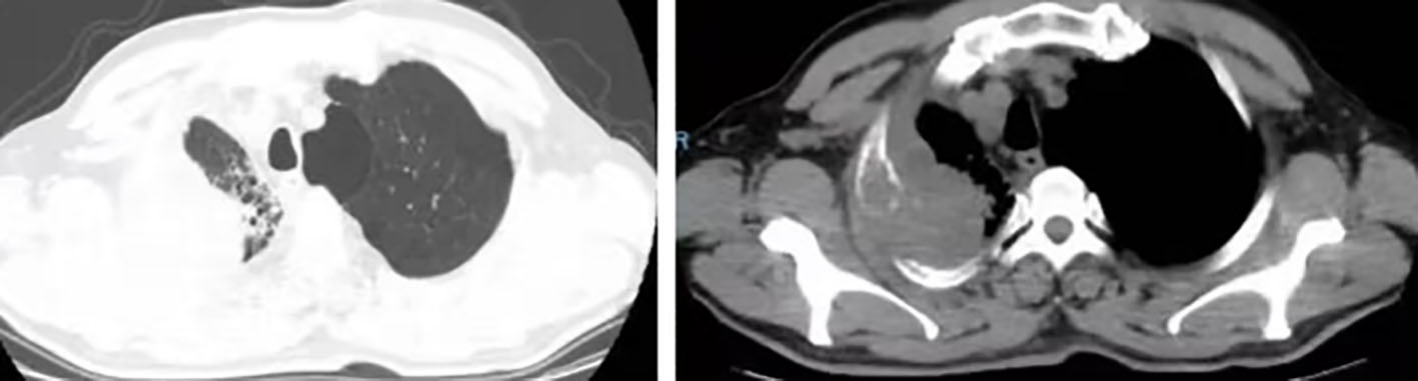
Four months after the operation, the tumor recurred and invaded the adjacent ribs.

A 63-year-old male presented to our hospital with blood-tinged sputum, dyspnea, and weight loss of approximately 5 kg. He had a history of heavy smoking, but no history of diabetes mellitus. There had no relevant family or medical history. Upon examination, he was conscious with vital signs within normal limits, except for a slightly decreased peripheral oxygen saturation of 92% on room air. Physical examination revealed decreased breath sounds in the right lung. Serum tumor marker assessment indicated elevated levels of neuron-specific enolase at 27.22 ng/mL and cytokeratin 19 fragment (Cyfra 21–1) at 3.43 ng/mL, whereas other tumor markers were within the normal ranges. Chest computed tomography (CT) revealed a mass in the right upper lobe, with a cavity (**Figure 1A**) and inflammation (**Figure 1B**). Despite antibiotic treatment, there was no improvement in the inflammation. Pathological tissue obtained via bronchoscopy confirmed the presence of poorly differentiated non-small cell lung cancer. Positron emission tomography/CT (PET/CT) showed the mass in the right upper lobe with a maximum standardized uptake value (SUV) of 44.67 (**Figure 2A**), and a right lung hilar lymph node metastasis could not be excluded (**Figure 2B**). No distant organ metastasis was observed (**Figure 2C**). The patient’s prognosis was initially considered poor. Surgery was performed after the exclusion of preoperative contraindications. The surgical plan included resection of the right upper and right middle lobes, as well as dissection of relevant lymph nodes, and was successfully executed as planned. Histopathological examination of the surgically resected specimen revealed undifferentiated tumor cells with eosinophilic cytoplasm, eccentrically located vesicular nuclei, prominent nucleoli, and frequent mitoses (**Figure 3A**), similar to SMARCA4-deficient thoracic sarcoma. The immunohistochemistry results were negative for Brahma-related gene 1 (BRG1) (**Figure 3B**), cytokeratin (CK) 5/6, and CK7. CK19 (**Figure 3C**) and CK (pan) cells (**Figure 3D**) were focally positive. Tumor cells were positive for integrase interactor 1 (INI-1) (**Figure 3E**), epithelial membrane antigen (EMA) (**Figure 3F**), and vimentin (**Figure 3G**). Additionally, the proliferation index Ki-67 (**Figure 3H**) was 90% in the tumor cells. The final diagnosis was SMARCA4-UT; no lymph node metastasis was found in the dissected lymph nodes, and the clinical stage was T3N0M0 stage IIB. The patient was initially satisfied with the treatment, but declined further chemotherapy and genetic testing. Although no recurrence was observed two months postoperatively, the tumor reappeared and invaded the adjacent ribs four months after the operation (**Figure 4**).

## Discussion

BRG1, a protein encoded by *SMARAC4*, is a crucial component of the SWI/SNF chromatin remodeling complex. Its inactivation and the loss of gene expression are associated with cancer (9). SMARCA4-UT, characterized by mutations in the *SMARCA4* gene that lead to inactivation and an undifferentiated or rhabdoid morphology, is often found in tumors of significant size. Almost all patients had a history of smoking and were clinically characterized by non-specific symptoms such as cough, dyspnea, and hemoptysis. SMARCA4-UT is often accompanied by loss of SMARCA2 (10). Immunohistochemistry typically shows a negative or low expression of cytokeratin and claudin-4 in SMARCA4-UT cells, whereas INI-1, vimentin, and Sox2 are usually positive (2). However, in some cases, SMARCA4 staining may show a significant decrease rather than complete negativity (2). Most cases are not suitable for surgery and usually require bronchoscopy or a fine-needle biopsy to obtain the pathological tissue, making diagnosis challenging. Next-generation sequencing (NGS) has been utilized for diagnosis but may miss 12.5% of SMARCA4-UT patients (11). Sanger sequencing, although accurate and low-throughput, is expensive and time-consuming for large-scale sequencing. Therefore, a combined approach using both immunohistochemistry and NGS is recommended for diagnosing SMARCA4-UT, with Sanger sequencing as a verification tool if needed (1).

SMARCA4-UT, previously referred to as SMARCA4-deficient thoracic sarcoma, has several unique features. It requires differential diagnosis from other tumors, such as malignant rhabdoid tumor (MRT), small cell carcinoma of the ovary hypercalcemic type (SCCOHT), and SMARCA4-deficient non-small cell lung cancer (NSCLC) (2). While SMARCA4-UT may share morphological similarities with MRT and SCCOHT, it is important to note that most patients with SMARCA4-UT are males with a history of smoking, frequently have TP53 mutations (2), and do not show a familial inheritance pattern (12). MRT is predominantly found in infants and young children (13), whereas SCCOHT primarily affects adolescents and young women (14). SMARCA4 mutations have been observed in 10% of NSCLC cases (15). SMARCA4-deficient NSCLC and SMARCA4-UT predominantly occur in heavy smokers. However, there are differences in tumor cell characteristics between SMARCA4-deficient NSCLC and SMARCA4-UT. In SMARCA4-deficient NSCLC, tumor cells display differentiated epithelial structures and strong expression of epithelial markers, whereas in SMARCA4-UT, tumor cells are poorly differentiated with scattered weak or negative expression of epithelial markers. Moreover, CD34, Sox2, and Syn are frequently expressed in SMARCA4-UT cells (16, 17). Therefore, for a young male heavy smoker presenting with a large undifferentiated or poorly differentiated chest tumor, clinicians should consider the possibility of SMARCA4-UT (1).

Radical surgery is the preferred method for treating malignant tumors; however, SMARCA4-UT often shows local invasion and distant metastasis, making only a few early stage patients eligible for surgery. Although initial surgery has been reported to extend overall survival (OS), the benefits may not be significant, and rapid tumor recurrence post-surgery is common (1). In the present case, adjuvant therapy was recommended based on the postoperative clinical staging, but the patient declined further treatment. The tumor had not recurred two months after surgery, but recurred and invaded the adjacent ribs four months postoperatively, with the patient still refusing adjuvant therapy. The short follow-up period and lack of data on adjuvant treatment in this specific patient scenario are limitations of this clinical case. Therefore, additional reports on surgically managed SMARCA4-UT cases are necessary to determine the most appropriate clinical stage for surgical intervention and a comprehensive treatment plan.

Chemotherapy is a commonly used treatment for tumors; however, no study has specifically reported the effectiveness of chemotherapy alone in patients with SMARCA4-UT (3). However, chemotherapy can enhance the immune environment, thereby boosting the antitumor effects of immune checkpoint inhibitors (ICI) (18, 19). Studies have indicated that the presence of tertiary lymphoid structures (TLS) in SMARCA4-UT is associated with a high response rate to ICI treatment. For instance, the 6-month non-progression rate was 40% in patients with TLS-positive SMARCA4-UT, as opposed to only 4.2% in all patients (7). Patients who receive first-line ICI therapy generally exhibit longer median OS and progression-free survival compared to those who receive ICI therapy in later lines or not at all (1). Some cases have demonstrated the effectiveness of immunotherapy as a neoadjuvant treatment, resulting in radical tumor resection and a complete pathological response (20). The efficacy of immunotherapy for SMARCA4-UT seems to be independent of programmed death ligand 1 (PD-L1) expression, which is often low or negative (21). Furthermore, the combination of anti-PD-1/PD-L1 antibodies and antiangiogenic agents demonstrated synergistic effects (22). Studies have indicated that atezolizumab, bevacizumab, carboplatin, and paclitaxel combination therapy is effective for treating SMARCA4-UT (23). However, the efficacy of immunotherapy may be compromised in cases in which high PD-L1 expression coincides with *KEAP1* mutations (23).

The most commonly mutated genes in SMARCA4-UT were *TP53*, *STK11*, *KEAP1*, and *KRAS* (24). Although *ALK* mutations have been reported in some cases, *EGFR* mutations typically observed in NSCLC have not been reported in SMARCA4-UT (5). Currently, there are no highly effective targeted drugs available.

Studies have suggested that HDACi, EZH2 inhibitors, and OXPHOS inhibitors may be effective in treating SMARCA4-UT. SMARCA4-UT is often accompanied by loss of SMARCA2. HDACi have been shown to restore SMARCA2 expression in various SMARCA2-deficient tumors, reduce tumor cell growth (9), enhance sensitivity to cisplatin, and restore sensitivity to PD-1 inhibitors. Patients with SMARCA4-UT have demonstrated the benefits of combination therapy involving HDACi, cisplatin chemotherapy, and PD-1 inhibitors (1). Targeted EZH2 inhibitors are currently under investigation for the treatment of cancers with SWI/SNF complex protein abnormalities such as SMARCA4-UT. Initial findings suggest that the therapeutic effects of EZH2 inhibitors are more pronounced when both SMARCA4 and SMARCA2 are mutated (25). Tumors harboring SMARCA4 mutations exhibit increased OXPHOS, and the use of OXPHOS inhibitors, such as IACS-010759, can reduce OXPHOS activity, leading to tumor cell death and decreased tumor progression (26). In phase I clinical trials involving solid tumors, 44% of patients treated with IACS-010759 achieved stable disease or partial response (27).

In conclusion, SMARCA4-UT is highly invasive, with most patients diagnosed at stage III or IV, precluding surgical intervention. Preoperative PET/CT is recommended to rule out distant metastases. Here, we report a rare case of SMARCA4-UT that met the criteria for surgical resection. Recent studies have indicated the effectiveness of immunotherapy for treating this condition. Furthermore, treatment with anti-angiogenic drugs, HDACi, EZH2 inhibitors, and OXPHOS inhibitors may also be beneficial for managing SMARCA4-UT.

## Data availability statement

The original contributions presented in the study are included in the article/supplementary material, further inquiries can be directed to the corresponding author/s.

## Ethics statement

The studies were conducted in accordance with the local legislation and institutional requirements. The participants provided their written informed consent to participate in this study. Written informed consent was obtained from the individual(s) for the publication of any potentially identifiable images or data included in this article.

## Author contributions

CY: Conceptualization, Data curation, Formal analysis, Investigation, Methodology, Project administration, Software, Supervision, Validation, Visualization, Writing – original draft, Writing – review & editing. Z-jL: Data curation, Formal analysis, Investigation, Methodology, Project administration, Resources, Software, Supervision, Validation, Writing – review & editing. CH: Conceptualization, Data curation, Formal analysis, Investigation, Methodology, Software, Supervision, Writing – review & editing. H-xY: Conceptualization, Data curation, Formal analysis, Funding acquisition, Investigation, Methodology, Project administration, Resources, Software, Supervision, Validation, Visualization, Writing – review & editing.
